# High-yield bioproduction of virus-free virus-like P4-EKORhE multi-lysin transducing particles as an antimicrobial gene therapeutic

**DOI:** 10.3389/fcimb.2025.1561443

**Published:** 2025-06-25

**Authors:** Robert Ramirez-Garcia, Antonia P. Sagona, Jeremy J. Barr, Alfonso Jaramillo

**Affiliations:** ^1^ School of Life Sciences, Faculty of Science, Engineering and Medicine, The University of Warwick, Coventry, United Kingdom; ^2^ School of Biological Sciences, Faculty of Sciences, Monash University, Melbourne, VIC, Australia; ^3^ Department of Computing, Faculty of Engineering, Imperial College London, London, United Kingdom; ^4^ Translation & Innovation Hub (I-HUB), Imperial College London, London, United Kingdom; ^5^ ACGTx, London, United Kingdom; ^6^ Institute for Integrative Systems Biology (I2SysBio), CSIC-Universitat de València, Paterna, Spain

**Keywords:** high-yield bioproduction, bioprocess engineering, virus-like particles, transducing particles, antimicrobials, gene therapeutics, A549-model of infection, P4-EKORhE

## Abstract

A description of the construction of the bioengineered P4-EKORhE and a comprehensive method for producing very high yields (up to 10^12^ particles per millilitre) enable the use of virus-like particles to transduce genetically encoded antimicrobials through a combination of synthetic biology and optimised upstream and downstream processing. The final product, a gene-delivered antimicrobial in the form of the multi-lysin cassette, is fully functional before and after packaging within P4-EKORhE particles. The antimicrobial activity of the multi-lysin cassette, characterised by its lysis proteins, was tested *in vivo* in both pure bacterial *Escherichia coli* cultures and a model of phage infection in co-culture with A549 immortalised human epithelial tissue cells. This work exemplifies several bioproduction methods and demonstrates how the virology of the P4 and P2 phages can be harnessed to establish a bioprocess for producing transducing particles at very high yields, avoiding contamination by the natural virus while maintaining the antimicrobial effectiveness of the final product.

## 1 Introduction

**Figure 1:**
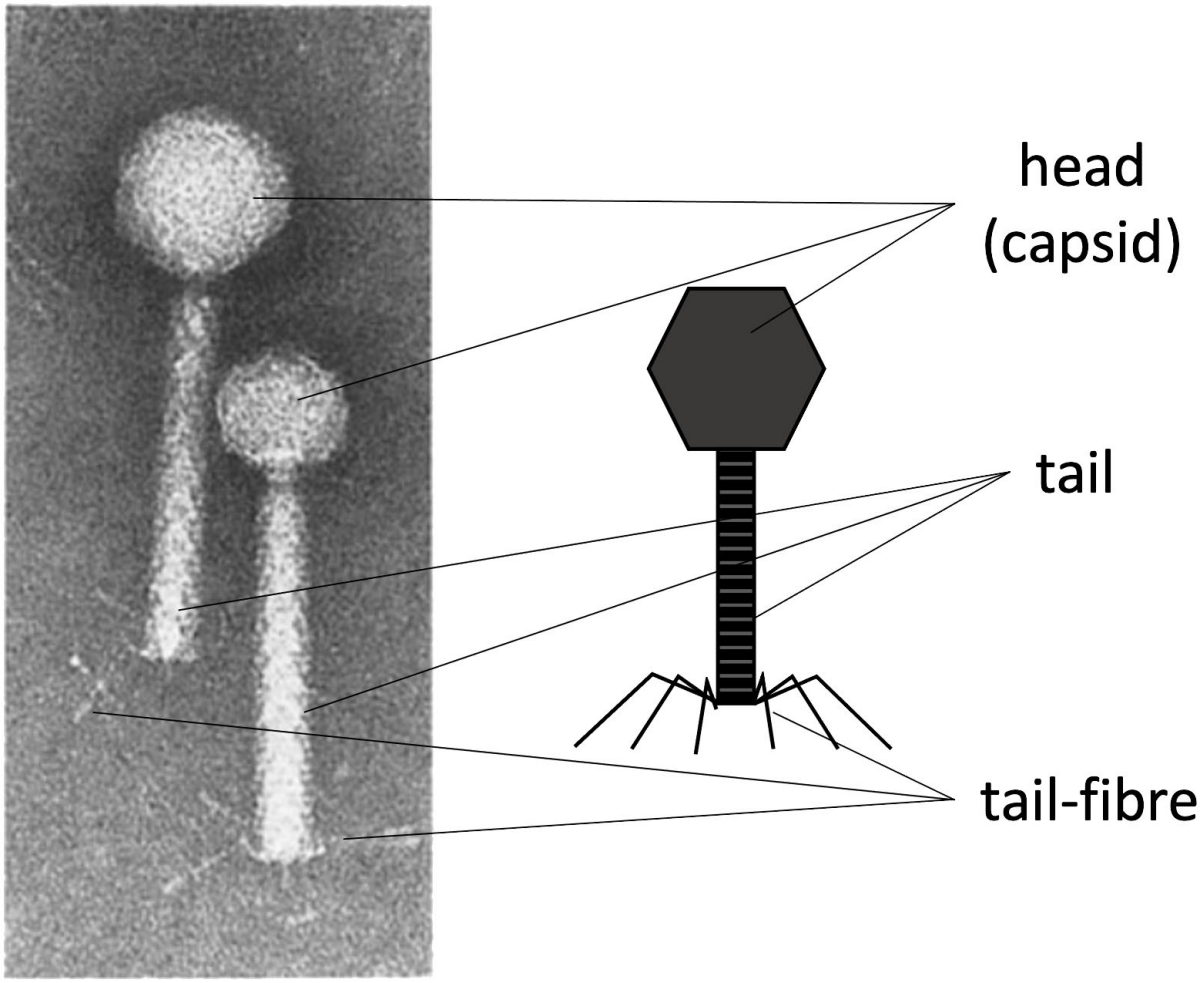
The characteristic protein structures in phages. *Left:* An electron microscope image of phage P2 (with a larger head) and P4 (with a smaller head). *Right:* A schematic labelling of the main protein structures in tailed phages. The capsid (head), the tail, and the tail fibres. The right side of the image was made by the author. Left side of the Figure was adapted with permission from (Goldstein et al., 1974), licensed under [6037081204133], [SPRINGER-NATURE].

Harnessing the natural mechanisms that phages use to repurpose their host bacteria’s metabolism to produce viral structural material and package viral DNA for self-replication and propagation is how transducing particles are produced. The most characteristic protein-made parts of phages are the capsid, which packages and protects the genetic material; a tail, which allows the genetic material to be ejected upon infection; and the tail fibres, which enable specific interaction with a particular bacterial host (Mourosi et al., 2022). For a general schematic of these basic structures, see **Figure 1**. These protein structures allow the phage to store, protect, and, upon infection, release the genetic material within the target bacteria. If these protein components can be produced while blocking the virus’s ability to package its own viral DNA, it is possible to produce a transducing particle that contains and delivers a heterologous genetic payload to a target bacterial host. This is particularly straightforward when using phages whose “cos site” has been identified, such as the *λ* or P2/P4 phages.

### 1.1 Virus-free virus-like particle production based on conditionally propagating P4 phage

**Figure 2:**
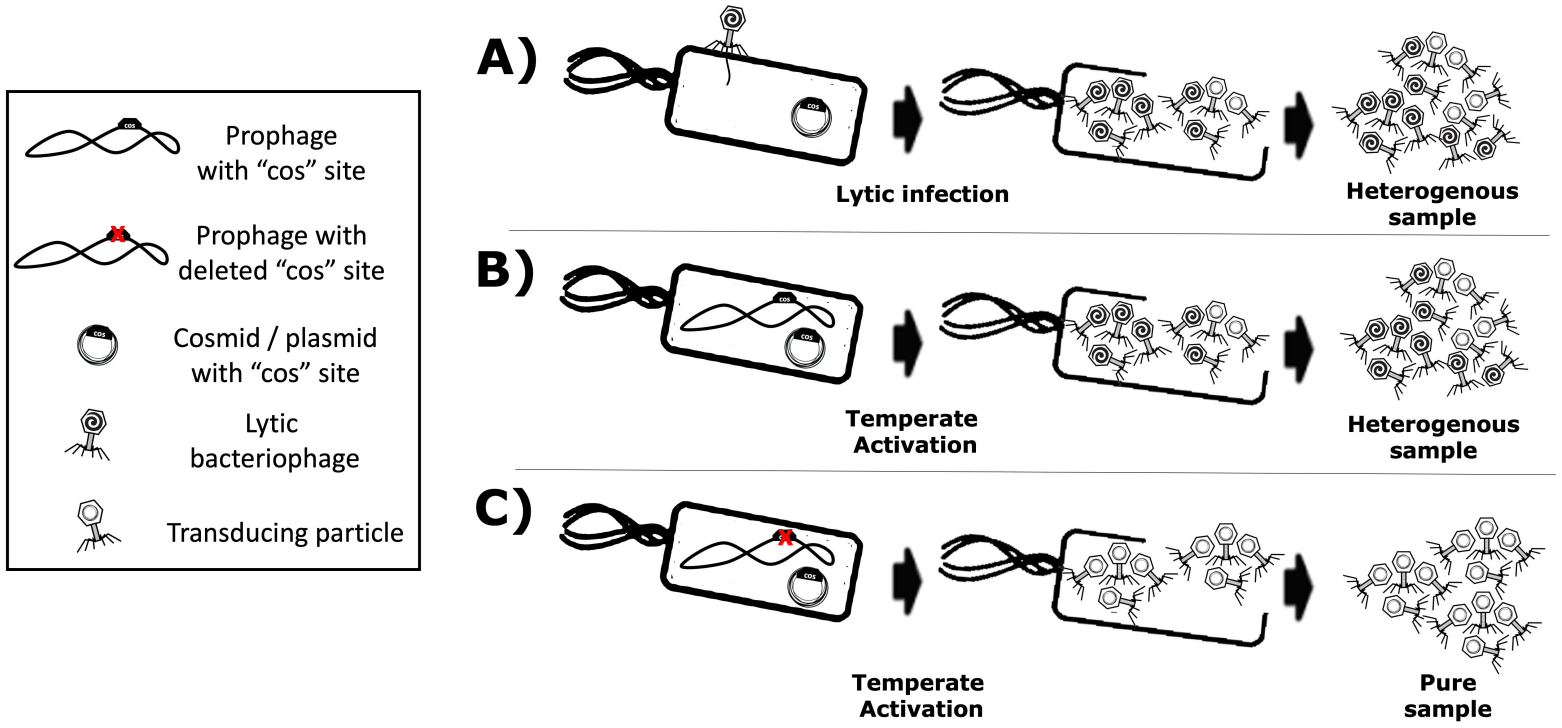
Production of transducing particles using lytic or temperate phages that use a cos site for packaging. Transducing particles are phage-based particles harbouring cosmids instead of phage genomes, which have been selected and maintained within host-producing strains. **(A)** Lytic phage production yields a mixture of phages and transducing particles. **(B)** Temperate phages with functional cos site also yield a mixture of phages and transducing particles. **(C)** A temperate phage with a *knocked-out* cos site yields a “virus-free” pure sample of transducing particles.

As described extensively in the literature (Goldstein et al., 1974; Gibbs et al., 1973; Six and Klug, 1973; Six, 1975), phage P4 is a helper-dependent phage that uses the late gene products of temperate phage P2 to encapsulate its own P4 double-stranded DNA. The P4 capsid is approximately one-third the volume of the P2 capsid (Inman et al., 1971) and harbours a genome also one-third its size (i.e. 11.624 versus 33.574 kb, respectively). As aforementioned, the P4 phage overexpresses the P2 prophage late genes (Six and Klug, 1973) without the P2 prophage being excised or replicated (Six and Lindqvist, 1971). Another characteristic of P4 is that, when a P2 prophage is not present in the host bacterial cell, P4 can still be maintained as a plasmid (Lindqvist and Six, 1971), which allows P4 to hijack a subsequent infection by a virulent P2 phage. Therefore, the viral cycle of phage P4 is a unique phenomenon in which one phage parasitises another to perpetuate itself, repurposing the gene products of the helper phage. While phage P2 constructs an icosahedral capsid (head) with T = 7 symmetry using the *gpN* capsid protein, the *gpO* scaffolding protein, and the *gpQ* portal protein, the presence of P4 drives these structural proteins into a smaller capsid with T = 4 symmetry. The regulation of this size transformation is facilitated by the P4-encoded protein *Sid*, which creates an external scaffold around the compact P4 procapsids (Dearborn et al., 2012). This capsid reduction restricts P2’s larger genome from propagating while favouring the packaging of the smaller P4 genome. The P4 phage viral cycle is described in the literature (Lindqvist et al., 1993). This interplay lets P4 exploit P2 resources while preserving its unique life cycle traits. Since P4 can be maintained as a plasmid and depends on P2 for conditional propagation (i.e. using P2’s late structural proteins for packaging and storage), it is feasible to use this phenomenon to produce transducing particles via P4’s conditionally propagating capability. Moreover, a minimal essential region of P4 has been described (Lin, 1984; Ghisotti et al., 1990). One can thus engineer a “conditionally propagating transducing particle” with a P4-minimal construct, here referred to as “P4-min”, measuring 7.190 kb and allowing the expression of the necessary gene products for fully functional P4 plus an additional capacity for a heterologous genetic sequence payload of up to 4.434 kb. This capacity parallels the full-size 11.624 kb of P4. A P4-min-based system can host a genetic device encoding antimicrobial-like molecules. This work illustrates a system that includes *the multi-lysin cassette*, a kanamycin-resistant marker, and a co-located P4 cos site spanning 4.151 kb. In this context, P4-min functions as a transducing particle capable of propagation only in the presence of P2. It thus reaches yields that are typical of natural phages (≥10^8^ cfu/mL). Here, a transducing particle is defined as a viral particle that carries a plasmid encoding heterologous or synthetic genetic material, along with a “cos site” acting as a “packaging signal”, instead of the phage’s own genome. **Figure 2** summarises how the P2/P4 phages can generate transducing particles.

The main disadvantage of using helper P2 phages for transducing particle production is the contamination of the lysate with wild-type phages, which are self-replicating and highly propagative. Such contamination can arise from non-adsorbed helper phage and the packaging of competing viral genomes. Because the virus’s DNA and the proteins needed for virion assembly are produced in the host cell, both the cosmid and the viral genome compete for packaging, yielding a mixture of transducing particles (plasmid/cosmid) and viruses (viral genome). **Figures 2A, B** show these scenarios. Using helper P2 prophages (i.e. lysogens) to produce transducing particles presents another drawback: the spontaneous induction of lysogenic cells into a lytic cycle occurs at a low frequency (Bertani, 1951). Temperate strains can be activated chemically (Endo et al., 1963) or by UV light (Baluch and Sussman, 1978; Berenstein, 1986), but each activator is phage-specific. For example, UV light can induce *λ* lysogens, but not P2 lysogens. In this regard, the epsilon (*ϵ*) gene product from natural P4 phage activates temperate P2 prophages (Liu et al., 1997). P2 (Lindqvist et al., 1993) “late genes” contribute to the production of the aforementioned phage structural proteins such as the capsid, the tail, and tail fibres (**Figure 1**) for its complete constitution as a virus (Kahn et al., 1991). In this light, transactivation of P2 late gene expression by P4 requires the P4 delta (δ) gene product and works even without P2 DNA replication (Halling and Calendar, 1990), benefiting P4 viral genome packaging over competing P2. Hence, P2 lysogenic strains can help produce phage-based transducing particles because inducers have been identified, and cosmid packaging can outcompete native viral genomes. Furthermore, temperate lysates become virus-free (**Figure 2C**) by *knocking out* the cos site of the temperate strain’s genome, ensuring that only foreign cosmids (with the intact cos site) get packaged. In this work, the P4-EKORhE, a virus-free virus-like transducing particle production system based on P4’s ability to hijack the P2 prophage’s late structural proteins, is shown to deliver a multi-lysin cassette as a gene-delivered antimicrobial.

### 1.2 Phage lysins as a gene-delivered antimicrobial

**Figure 3:**
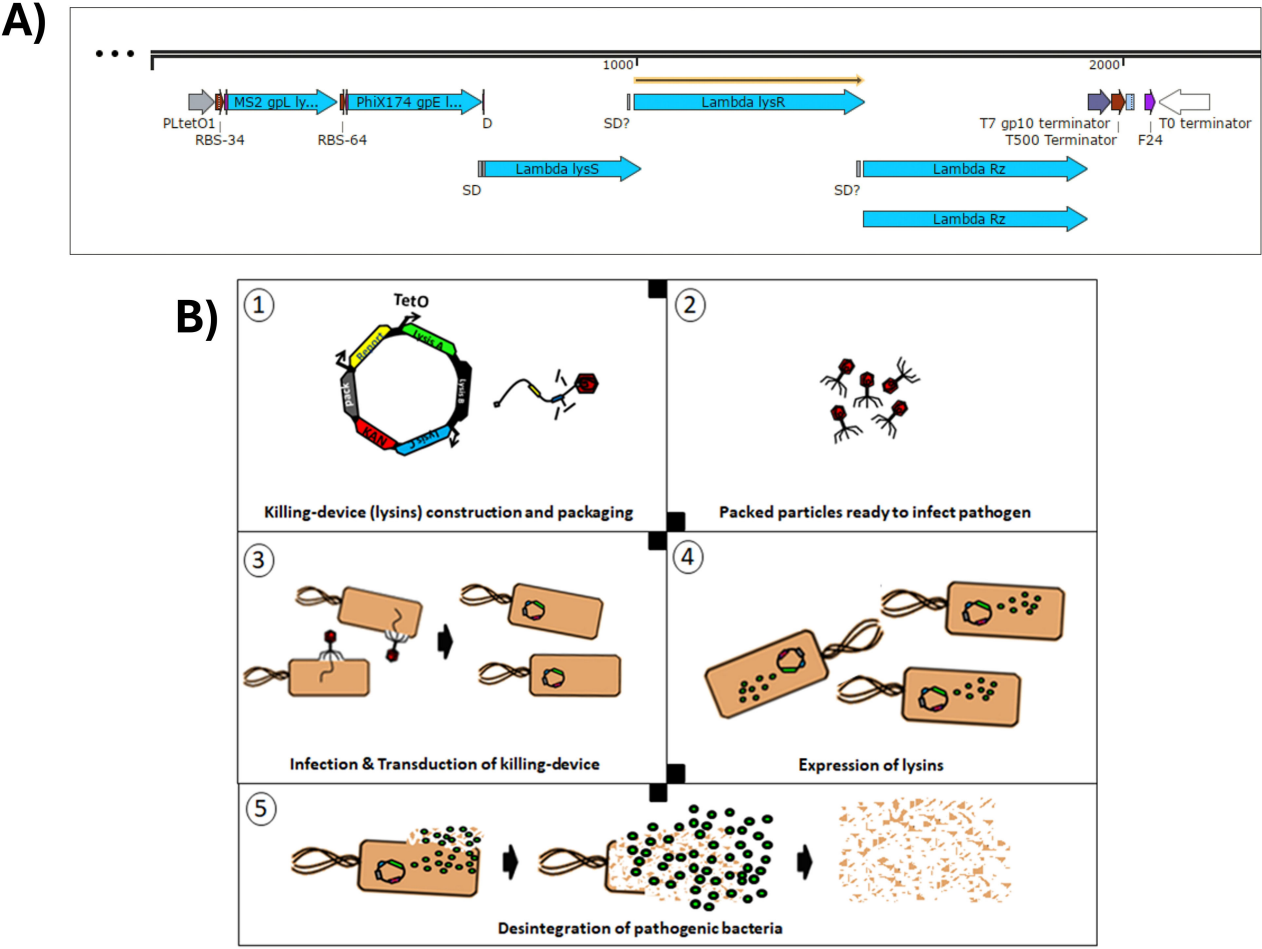
Conceptual scheme of the P4-EKORhE multi-lysin antimicrobial. **(A)** The multi-lysin cassette harbouring all lysins and lysis accessory proteins. The different encoded lysis and lysis accessory proteins used to construct the multi-lysin cassette. A study of the functionality of these circuits is presented in **Figure 4**. The “SD” and “SD?” labels contained within the construct are predicted “Shine–Dalgarno” sequences that correspond to the multi-cistronic nature of the *λ* phage lysis operon n (*lysS*, *lysR*, and *Rz*) and the likely presence of unidentified *cryptic promoters*. **(B)** Conceptual scheme of the P4-EKORhE multi-lysins as a genetically encoded antimicrobial therapeutic.

**Figure 4:**
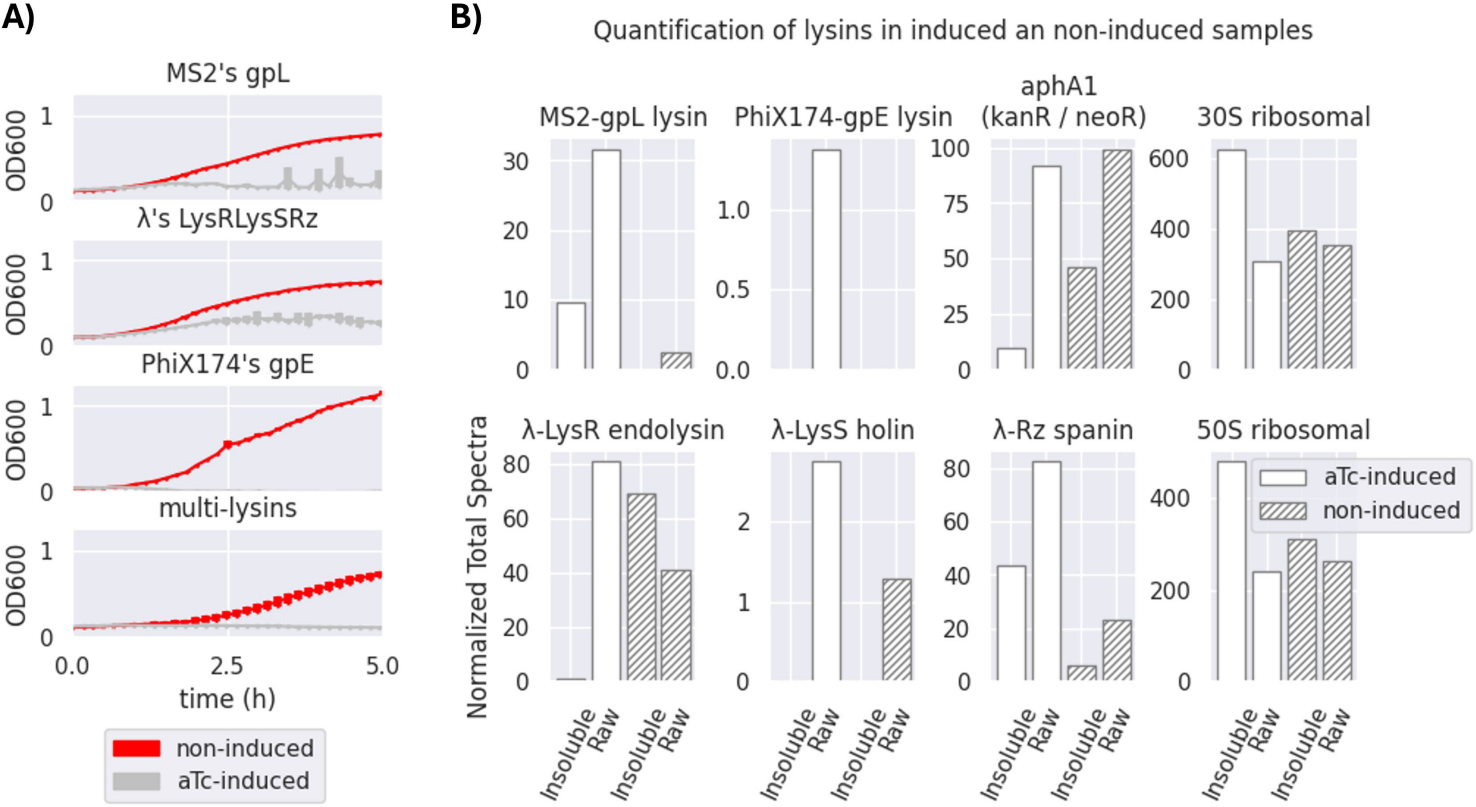
Effectiveness of a transformed multi-lysin cassette. **(A)** Optical density (OD_600_) measurements of bacterial survival profiles in pure bacterial cell cultures of *Escherichia coli* DH5*α* Z1 showing the antimicrobial effect of the multi-lysin cassette (the bottom-most plot) and each of its contributors (MS2’s gpL, *λ*’s lysis cassette, and PhiX174’s gpE; the top-three-to-bottom plots). The red lines represent the bacterial cells harbouring the multi-lysin cassette that have not been induced with anhydrous tetracycline (aTc). The silver lines represent bacterial cells harbouring the lysin cassette that have been induced with anhydrous tetracycline at the start of the culture. * A second replica is available in **Supplementary Figure S1** of **Supplementary Section SS2** for Replicas. The thickness of each line represents variability among the “n” samples using the standard deviation, where n = 3. **(B)** Normalised Total Spectra (NTS) obtained from a mass spectrometry (MS) analysis of four different samples (two induced and two non-induced, raw and insoluble phases) of bacteria containing the multi-lysin cassette. The Normalised Total Spectra include data that compute the peptides found with over 95% probability that belong to each of the lysin sequences studied. aTc-induced: The protein extract comes from bacteria for which cosmids containing the multi-lysin cassette have been activated by adding aTc (anhydrous tetracycline) to the culture media. Non-induced: The protein extract comes from bacteria for which cosmids containing the multi-lysin cassette have not been externally induced with aTc. Insoluble fraction: Protein fraction extracted from the pellet of the lysed cells. Raw fraction: Protein fraction extracted from samples containing both the supernatant and the pellet of lysed cells. For more details, see **Supplementary Section SS3**.

**Figure 5:**
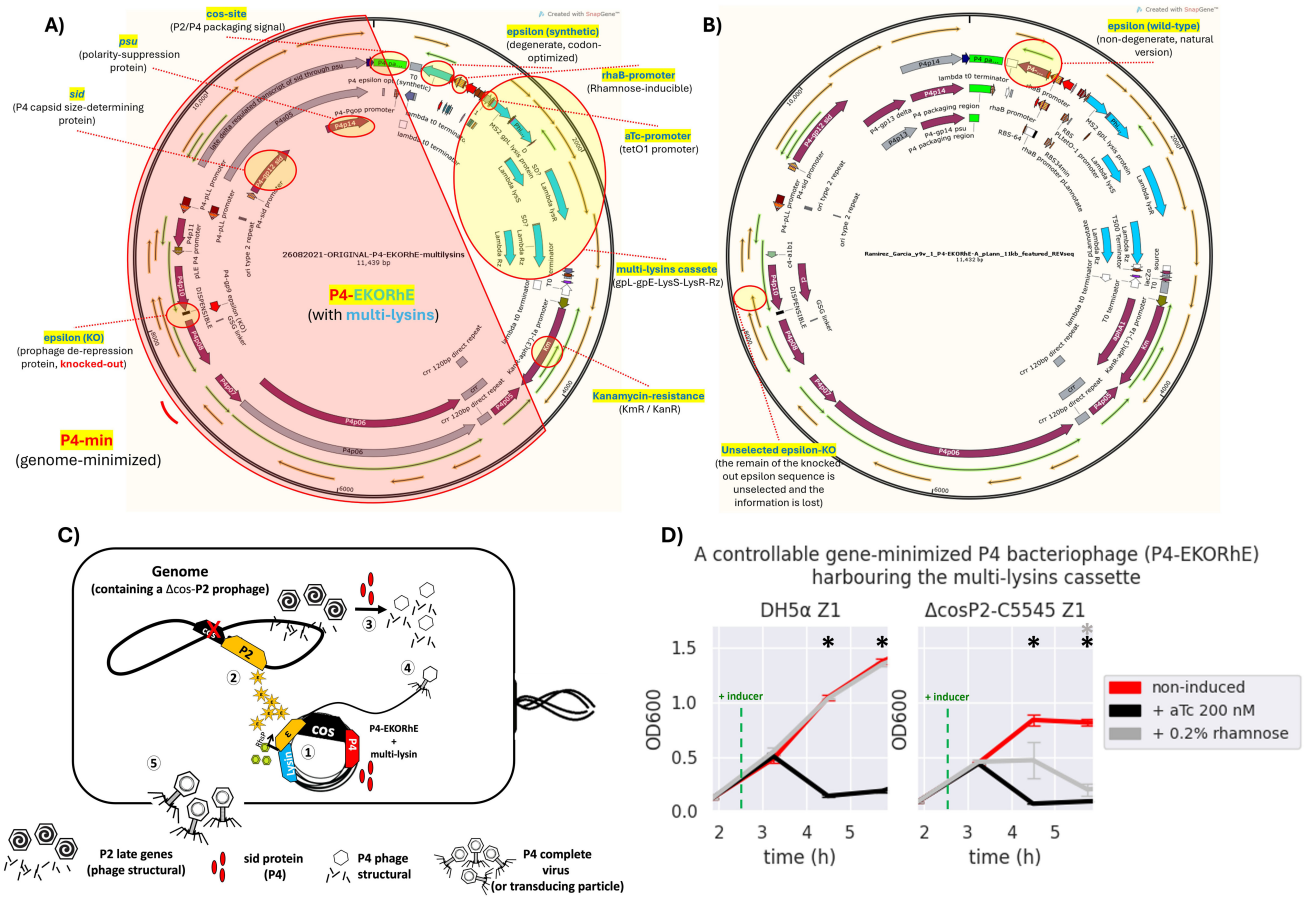
The bioengineered P4-EKORhE harbouring the multi-lysin cassette. **(A)** A kanamycin-selectable cosmid of 11,439 bp containing a genome-minimised P4 phage (P4-min, highlighted in red) that has a knocked-out version of the epsilon (*ϵ*) gene; an alternative functional synthetic, codon-optimised, and degenerate version of the *ϵ* gene; and the multi-lysin cassette harbouring the three systems of lysis from MS2, PhiX174, and *λ* phages. **(B)** The same cosmid after a few passages loses the dysfunctional *knocked-out wild-type* version of the *ϵ* gene, while the synthetic degenerate version gets restored to the sequence observed in the wild-type *ϵ* protein commonly seen in the natural P4 phage [see red circles for “unselected epsilon-KO” and “epsilon (wild-type)”]. The stable version of the P4-EKORhE is now 11,432 bp. Fully featured sequences of panels A and B can be found in **Supplementary Sections SS8.5** and **SS8.6**, respectively. **(C)** A conditionally propagating and rhamnose-inducible P4 phage as a transducing particle. (1) The P4-EKORhE is replicated as a cosmid harbouring the multi-lysin cassette and expresses the *Sid* protein upon activation. (2) A Δcos version of the P2 prophage is inhibited by the activity of P4 but activates its late genes, with the delta (*δ*) gene product, which are the necessary structural proteins for P2. Additionally, the rhamnose-inducible *ϵ* gene product activates the self-replication of the Δcos version of the P2 prophage. (3) The *Sid* protein from P4 converts P2 heads into smaller P4 heads, thus further compromising P2 propagation. (4) P4 structural proteins package the P4 cosmid harbouring the multi-lysin cassette (i.e. or any other engineered circuit) or any other accessory cosmid. **(D)** Dynamics of the activation of the P4-EKORhE multi-lysin cosmids with inducer, aTc or rhamnose, in strains of *Escherichia coli*, transformed on *E. coli* DH5*α* Z1, or the engineered lysogen Δcos:TriR-P2-c5545 Z1. The sample size (n) used is n = 3; *p-value < 0.05, measured at the data points used after the addition of the inducer, using a standard t-test using SciPy libraries (see General Methods SS6.1).

**Figure 6:**
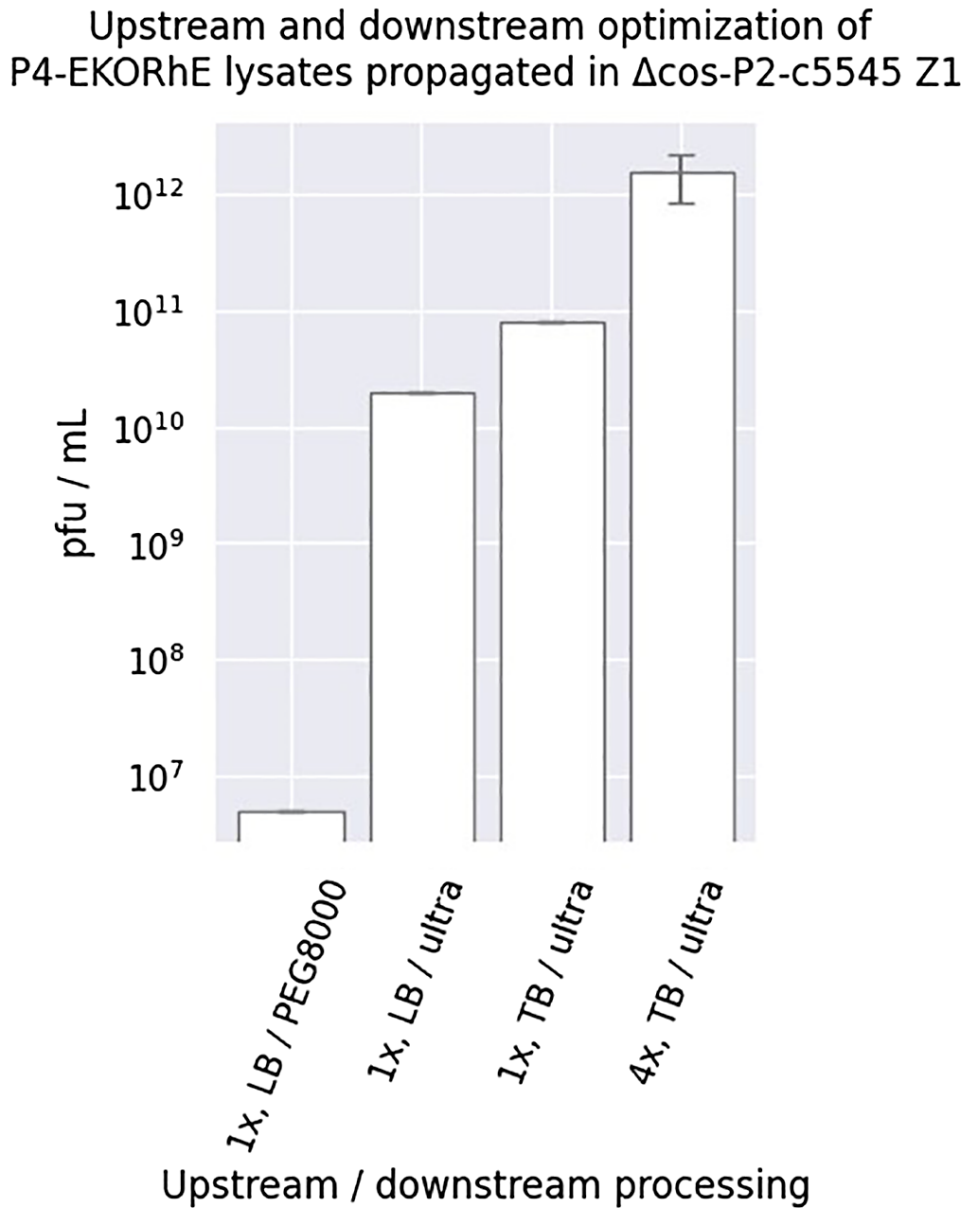
Upstream and downstream optimisation of P4-EKORhE. Different yields were obtained when using P4EKORhE according to the upstream and downstream optimisation. In regard to upstream optimisation, lysates were produced using either one or four enrichment cycles (i.e. 1× or 4×) using Luria–Bertrani (LB) or Terrific Broth (TB) broth. In regard to downstream optimisation, lysates were concentrated using either 10% PEG8000 or molecular weight cut-off ultrafiltration. The plaque-forming units are taken from the plates shown in **Supplementary Figure S4**. The sample size (n) used is n = 1, except for “4×, TB/ultra”, which has n = 3.

**Figure 7:**
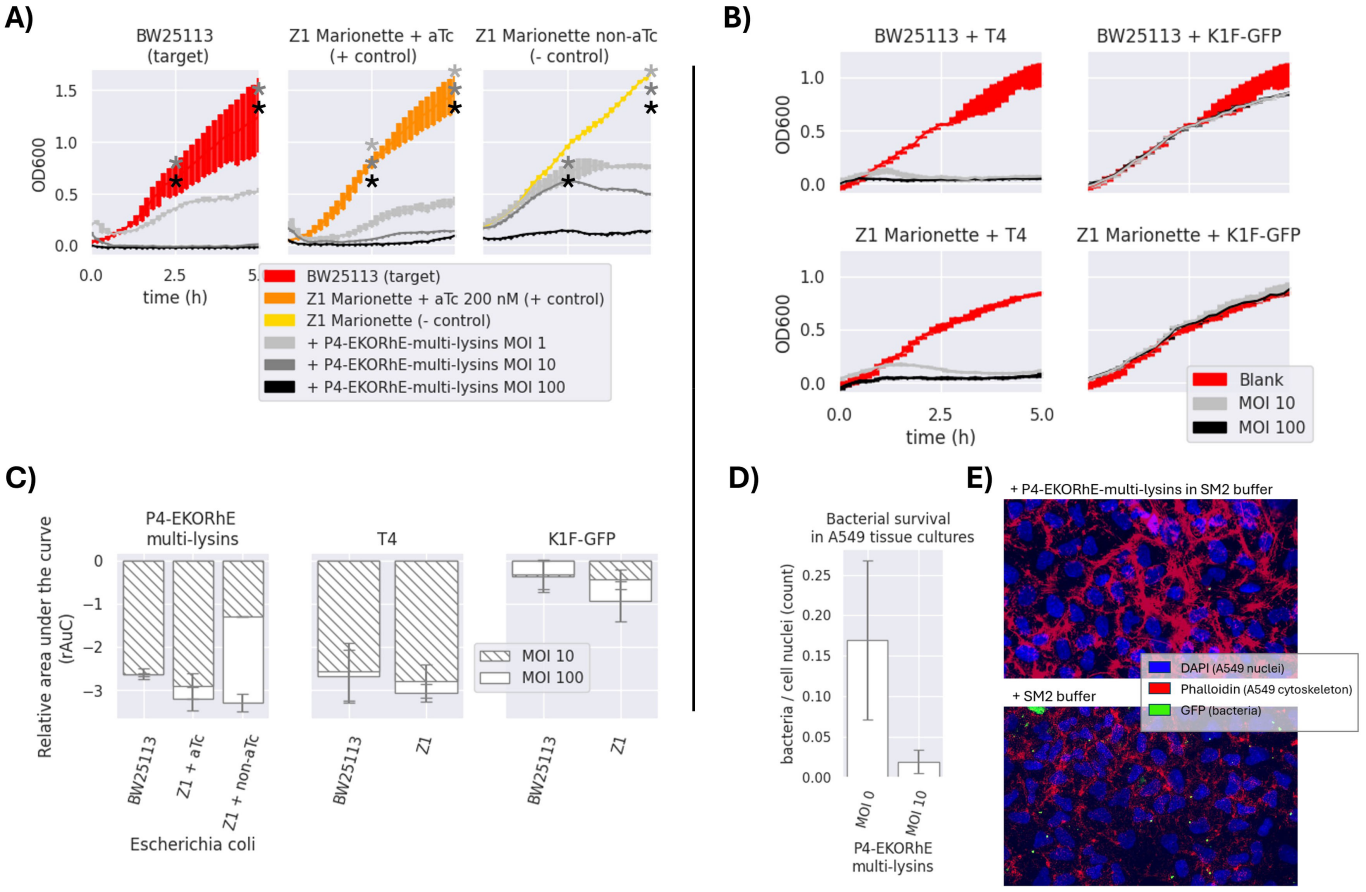
**(A)** Antimicrobial activity of P4-EKORhE multi-lysins. Optical density measurements of the target and control *Escherichia coli* strains, i.e. BW25113 and Z1 Marionette, respectively, when exposed to P4-EKORhE multi-lysin particles at different multiplicities of infection (MOIs). No selection antibiotics were used for the realisation of these experiments. **(B)** Antimicrobial activity of natural enterobacteriophages to their respective hosts. Antimicrobial activity of enterobacteriophages to their respective target and control *E. coli* strains, i.e. BW25113 and Z1 Marionette, when exposed to natural enterobacteriophages containing a replicative virus at different MOIs. For a replica of the same set of experiments, see **Supplementary Figure S2** in **Supplementary Section SS2** for Replicas. The thickness of each line represents variability among the “n” samples using the standard deviation, where n = 3. **(C)** Relative area under the curve of P4-EKORhE multi-lysins, T4, and K1F-GFP at MOIs of 10 and 100 on *E. coli* strains BW25113 (target) and Z1 Marionette (+/− control). rAuC(MOI = 0) = 0 ± 1. The sample size (n) is n = 2 from the results obtained in the two different replicas in **Figure 7** and **Supplementary Figure S2**, which contains n = 3 for each measure of time (total n = 6). The rAuC has been integrated from 0 to 5 hours in all cases. **(D)** The differences in bacterial count following treatment with P4-EKORhE harbouring the multi-lysin cassette on A549 cell tissue at an MOI of 10 versus with a blank solution (MOI = 0) of SM2 buffer, following an immunohistochemistry fluorescence image analysis. **(E)** Immunohistochemistry fluorescence image analysis on a sample infected with GFP-fluorescent bacteria treated with P4-EKORhE harbouring the multi-lysin cassette versus the blank solution of SM2 buffer. The sample size (n) used is n = 7 for the control, while n = 6 for the target. The p-value for the difference between the two samples is p = 0.31. The images used to count for the results shown in panel D can be found in **Supplementary Section SS5** for immunohistochemistry imaging. * means p-value < 0.05.

The multi-lysin cassette is a combination of genetically encoded lysins designed for delivery via transducing particles. Phage lysins and lysis accessory proteins (e.g. holins and spanins) are natural effectors that disrupt bacterial membranes or cell walls to release phage progeny. Because these proteins typically target bacterial membranes and cell walls, they resemble membrane-disrupting antibiotics by compromising bacterial viability (Monnard and Walde, 2015). Phage satellite P4 host range is restricted to Enterobacteriaceae (Gjorgjevikj et al., 2025). In this sense, *Escherichia coli* K12 is used as a target because of its low biosecurity risk and its role as a model for more pathogenic strains, including *E. coli* K1, which can cross the blood–brain barrier in neonatal meningitis and is a known uropathogen (Møller-Olsen et al., 2018). Combining multiple lysins and lysis accessory proteins can strengthen antimicrobial effects, as seen previously with extracellular lysins (Budič et al., 2011). Here, however, the lysins are genetically encoded and expressed intracellularly in the target bacterium. Both MS2 gpL and PhiX174 gpE single-gene lysis proteins and the *λ* lysis system (LysS, LysR, and Rz) are used within a single cassette (**Supplementary Section SS4.5**). **Figures 3** and **4** illustrate how these components function synergistically. In this work, a rhamnose-inducible, controllable, and conditionally propagating phage, the P4-EKORhE (**Figure 5**), is utilised to encode within its genetic cargo capacity, which is only limited by the *headful* size of its original genome, the multi-lysin cassette, as a means to test the use of a gene-delivered antimicrobial. Using a high-yield bioproduction protocol (**Figure 6**) of transducing particles encoding the multi-lysin cassette, these particles are then characterised for its effectiveness as an antimicrobial (**Figure 4**), using both pure bacterial cell cultures (**Figure 7C**) and a human model of phage infection in co-culture with A459 immortalised human epithelial cells (**Figures 7D, E**) used to corroborate the effectiveness of gene-delivered antimicrobials using P4-EKORhE in a more challenging biological environment. In this light, one of the novelties of the proposed approach is applying the concept of searching synergies among different lysins and lysis accessory proteins that act as they are meant to act: intracellularly, taking advantage of transduction as a gene delivery vehicle for the intracellular expression of effector proteins (i.e. the lysins and lysis accessory proteins, showcased in this work). **Supplementary Figure S12** shows the first version of a functional cosmid, encoding the multi-lysin cassette, with a strong anhydrous tetracycline (aTc)-inducible promoter. **Figure 3** shows a conceptual scheme of how the P4-EKORhE multi-lysins are employed as an antimicrobial therapeutic.

## 2 Results

### 2.1 The multi-lysin cassette

**Figure 4** demonstrates the effectiveness of each lysin and its combined bactericidal activity. PhiX174 gpE is the strongest contributor to multi-lysin synergy. **Figure 4** shows that the activation of lysis results in an antimicrobial effect, as observed over a 5-hour period. This (together with results demonstrated in **Figures 5**, **7**) suggests that the multi-lysin cassette is a strong alternative to natural phages.

The controllable expression of the multi-lysin cassette relies on the tightly conditional expression of lysins upon the addition of aTc, which hampers the structure of the tetR repressor. In this regard, there is an ample variety of promoters available that are of diverse strengths in *E. coli* (Meyer et al., 2019). In this work, a strong aTc-repressible promoter (Debowski et al., 2013), for the tight and strong induction of the multi-lysin cassette, and a weaker, rhamnose-inducible promoter (Wegerer et al., 2008; Kelly et al., 2018), to control P4-EKORhE transducing particle production, were used. The weak rhamnose-inducible promoter has a low expression profile even during induction, compared with tetO1, which helps express *ϵ* at very small levels and mitigates the potential leakage by weakness rather than tightness (a particularity of the tetO1 promoter that was decisive for its use in lysin expression).

In **Supplementary Figure S7**, the function of the tetO1 promoter in conjunction with the tetR repressor is exemplified (Debowski et al., 2013). The rhamnose-inducible promoter, instead, works by a different mechanism related to glucose availability (Kelly et al., 2018). The “tetO1 promoter” (Debowski et al., 2013) is only repressed if utilised in “Z1 cells” containing the “tetR repressor”. The Z1 cells used in this work were *E. coli* strains, such as DH5*α* Z1 or Marionette Z1. When the tetO1 promoter is present in bacterial cells not containing the Z1 cassette (e.g. the BW25113 cells), the promoter is not repressible; therefore, constitutive gene expression of the coding sequences downstream of the tetO1 promoter occurs. Since genetically encoded antimicrobials are to be delivered via bacteriophage-like transducing particles, it is important that during the production of transducing particles and the packaging of genetically encoded antimicrobials, a strong enough promoter be used (e.g. the tetO1 when used in Z1 cells). As shown in **Supplementary Figure S7**, the tetO1 promoter would repress the encoded genetic circuit in “production cells” harbouring the tetR repressor, which is what can be observed in non-induced samples in **Figure 4**. Conclusively, these can be safely used to produce high titres of transducing particles ready to package cosmids (i.e. plasmids with a packaging signal) containing an aTc-inducible lysin cassette without the risk of activating it, as is also demonstrated in **Figure 5D**.

Given that the multi-lysin cassette is delivered via P4-EKORhE transducing particles, a sufficiently strong promoter is essential during the production and packaging of this genetically encoded antimicrobial (e.g. the tetO1/aTc-inducible promoter used in Z1 cells). The tetO1/aTc-inducible promoter (PLtetO1 in **Figure 3A**) represses the encoded genetic circuit in “production cells” harbouring the tetR repressor. These cells can safely produce high titres of transducing particles ready to package P4-EKORhE cosmids encoding the multi-lysin cassette without the risk of premature activation. **Supplementary Figure S12** shows the first functional version of the multi-lysin cassette under the tetO1 promoter, also used in the high-yield P4-EKORhE that appears in **Figure 5**. The multi-lysin cassette in this work combines three lysis systems from the PhiX174, MS2, and *λ* phages (Garrett and Young, 1982; Didovyk et al., 2017; Young et al., 1979; Hutchison and Sinsheimer, 1966; Atkins et al., 1979) (**Supplementary Section SS4.5**), expressed under a single promoter and sized appropriately for packaging in P4-EKORhE.

As seen in **Figure 4**, all three systems of lysins (MS2, *λ*, and PhiX174) seem to contribute noticeably to the multi-lysin cassette, with PhiX174 gpE being the most outstanding contributor. Proteomics analysis of lysins/lysis accessory proteins in induced and non-induced samples of the multi-lysin cassette is shown in **Figure 4B**. Four samples were analysed: two induced with aTc (both soluble and insoluble fractions, as well as insoluble-only fractions) and two non-induced samples (labelled as non-induced in the figure) also for both raw and insoluble fractions. **Figure 4** shows the Normalised Total Spectra (NTS) of peptides corresponding to the lysins and lysis accessory proteins targeted in this work, with kanamycin-resistant aphA1, 30S, and 50S ribosomal proteins acting as positive controls for cosmid-borne and genome-borne expression. **Supplementary Section SS3** further details peptide coverage for these MS samples (**Supplementary Figures S5, S6**). **Figure 4** confirms the presence of cryptic promoters and Shine–Dalgarno (SD) sequences in the *λ*-lysis cassette (**Figure 3A**; **Supplementary Figure S8**). Peptides from *λ*-lysis cassette were detected in both induced and non-induced samples, with an important increase observed upon activation with aTc.

### 2.2 Construction of the P4-EKORhE

The virus-free, virus-like, conditionally propagating P4-EKORhE (a latinised transcription for P4-ϵKO-Rhϵ), which literally stands for “P4 epsilon (ϵ) knock-out rhamnose-inducible epsilon (ϵ)”, is a bioengineered rhamnose-inducible P4-based system for the high-yield bioproduction of transducing particles presenting itself as a genome-minimised P4 (P4-min) phage with its *ϵ* gene *knocked out*, and a degenerate, codon-optimised *ϵ* expressed elsewhere under the control of a rhamnose-inducible promoter (**Figure 5**), which is in this work used to harbour the multi-lysin cassette (**Supplementary Figure S12**).

During construction, P4-min (from P4p05 to P4s04, **Figure 5A** or **Supplementary Section SS8**) was PCR-amplified and cloned together with the multi-lysin cassette to confer a selection marker (kanamycin resistance). Following successful selection with kanamycin, the genome-minimised P4 (P4min) was further amplified by PCR to produce a *knock-out* in the native *ϵ* gene coding sequence and to insert a codon-optimised *ϵ* that cannot recombine with the native gene. The whole *ϵ* gene, the target for the *knock-out*, was not fully removed; instead, a minimal deletion was introduced to avoid the potential disruption of P4 functionality. Ultimately, a final mutant was obtained with a stably *knocked-out ϵ* gene, and a construct was inserted containing a degenerate version of the *ϵ* gene downstream of the cos site (located away from the original position of the *ϵ* gene) under a rhamnose-inducible promoter. Therefore, P4-EKORhE differs from a natural P4 phage because it is a cosmid that 1) remains genome-minimised, 2) carries a selection marker, and 3) incorporates two *ϵ* gene sequences (with the native epsilon being non-functional and the inserted version fully functional), thereby preventing recombination. **Figure 5** displays the final construct of P4-EKORhE harbouring the multi-lysin cassette. The P4-EKORhE genetic sequence was confirmed by analysing trace sequences from the assembled P4-EKORhE (**Figures 5A, B**), and the full sequence of the “stabilised form” of P4-EKORhE was obtained via whole-plasmid sequencing (**Figure 5B**); both sequences appear in **Supplementary Sections SS8.5** and **SS8.6**. **Figure 5C** illustrates a scheme that shows how the controllable, gene-minimised P4-EKORhE produces transducing particles. The figure demonstrates that the system activates upon induction because P4-EKORhE encodes a synthetic *ϵ* coding sequence downstream of the rhamnose-inducible promoter.

### 2.3 High-yield bioproduction of transducing particles with P4-EKORhE

**Figure 8:**
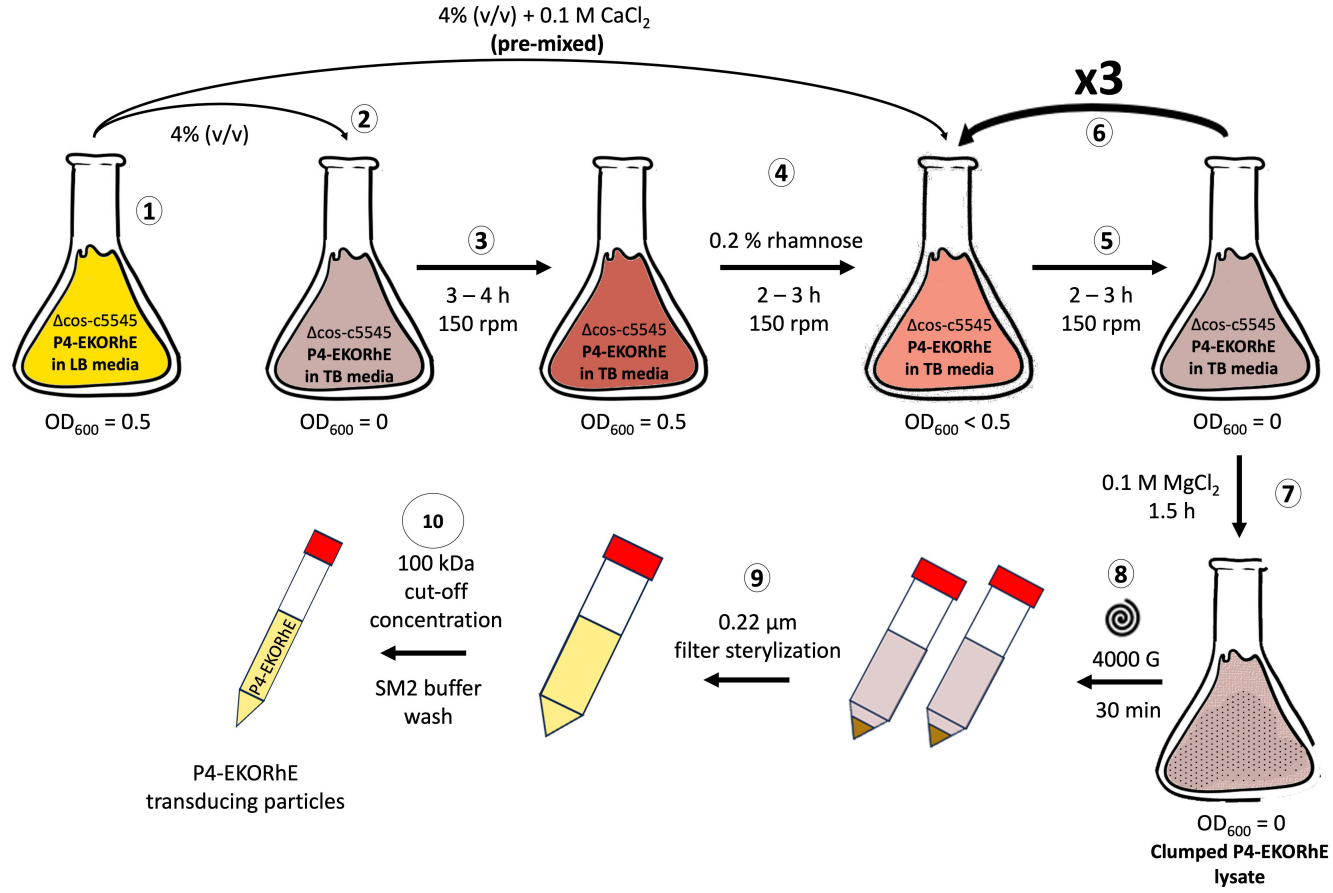
High yield P4-EKORhE-based transducing particle production protocol. Steps (1) to (10): (1) A flask containing a Δcos:TriR-P2-c5545 Z1 culture maintaining the P4-EKORhE cosmid is grown in Luria–Bertrani (LB) media until reaching exponential phase (i.e. OD_600_ = 0.5). (2) A 4% (v/v) sample of the exponentially growing LB culture is transferred into a flask containing Terrific Broth (TB) media. (3) The TB culture is grown to an exponential phase. (4) The TB culture is supplemented with 0.2% (w/v) rhamnose for the activation of the P2 prophage to enable the production of P4 particles, and a pre-mixed solution of exponentially growing cells with CaCl_2_ is prepared to reach a concentration of 0.1 M. (5) The TB culture is grown for 2–3 hours to accumulate lysed cells containing a proportion transducing particles. (6) The TB culture is re-supplemented at a 4% (v/v) with the initial exponentially growing LB culture of Δcos:TriR-P2-c5545 Z1 and re-balanced to maintain 0.1 M CaCl_2_ and 0.2% (w/v) rhamnose for as many cycles as necessary (i.e. from 5 to 6). (7) The TB culture is supplemented with 0.1 M MgCl_2_ and further grown for 1.5 hours or until clumps are observed. (8) The clumped lysate is pelleted at 4,000 *g* for 30 minutes to separate cell debris. (9) The supernatant is filter-sterilised. (10) The lysate is concentrated using a 100-kDa molecular weight cut-off ultrafiltration column and replaced with SM2 buffer to obtain a cleaned-up final lysate of P4-EKORhE transducing particles.


The use of the P4-EKORhE system for transducing particle production benefits from the controllable feature of its rhamnose-inducible synthetic *ϵ* cassette. However, optimising the bacterial cell culture conditions upstream, together with downstream processing that preserves particle integrity and maximises yield, remains essential. **Figure 6** highlights the improvement obtained when the literature standard PEG8000 phage purification is replaced with an ultrafiltration protocol (Bonilla et al., 2016), and the culture medium is switched from Luria–Bertrani (LB) to Terrific Broth (TB). The non-propagating nature of transducing particles when used on target strains requires high concentrations sufficient to observe a bacterial count decrease on the optical density measurements (**Figure 7**) that usually start at the range of 10^7–^10^8^ bacterial cells per millilitre when the optical densities are still close to zero. The controllability of P4-EKORhE enables the implementation of a specialised bioprocess in which continuously enriched phage lysates in a fed-batch fashion can be supplemented with growing cultures in parallel, successive batches of fresh Δcos-P2-c5545 Z1 cells harbouring P4-EKORhE (as shown in steps 5 and 6 in **Figure 8**). The process shown in **Figure 6** yields at least 10^9^ infectious particles per drop of 10 μL used for the spot assay (**Supplementary Figure S4**), which translates to a lysate titre of approximately 10^12^ pfu/mL (i.e. a transduction capacity of 10^12^ cfu/mL), according to the formula below:

$$\begin{array}{c} titer=dilution_{lysate}\cdot count_{plaques}\cdot volume^{-1}{\;}_{sample}\\ =pfu\cdot mL^{-1}\;or\;cfu\cdot mL^{-1} \end{array}$$

Therefore, **Figure 6** shows how the P4-EKORhE system can be optimised to yield 10^12^ cfu/mL of transducing particles by performing up to four enrichment cycles, utilising a nutrient-rich TB and the ultrafiltration protocol as a physical means of separation and concentration (**Figure 8**) of a high-yield virus-free lysate of transducing particles without P2 phage contamination (**Supplementary Figures S3, S4**).

### 2.4 Transduction of the multi-lysin cassette with P4-EKORhE

In **Figure 7A**, the antimicrobial potential of the multi-lysin cassette delivered by P4-EKORhE particles is demonstrated. The optical densities (OD_600_) of a transduced bacterial culture were measured over 5 hours and compared with natural phages T4 and K1F using the same target bacteria (**Figures 7B, C**).

**Figure 7** shows that P4-EKORhE multi-lysin particles exert an antimicrobial activity proportional to the multiplicity of infection (MOI). In particular, effective activity was observed at an MOI of 10. In the positive control, where the tetR repressor was inactivated with 200 nM aTc, the antimicrobial response was distinctive compared to the negative control, in which active tetR repressor significantly inhibited lysin expression. The target strain’s response matched or exceeded that of the positive control over the 5-hour measurement period.

### 2.5 The P4-EKORhE on A549 tissue cultures as a model of phage infection

Experiments measuring optical cell densities in pure bacterial cultures were used as an initial assessment of transducing particle effectiveness, alongside natural phages. However, to develop a potential antimicrobial therapeutic, it is valuable to replicate experimental conditions similar to real scenarios. Therefore, an infection model was employed that uses A549 human lung epithelial immortalised cells to further assess the capacity of P4-EKORhE encoding the multi-lysin cassette to function as an antimicrobial in a more complex environment.

In **Figure 7D**, the ratio of bacteria to nuclei is demonstrated from images taken in two experiments: one where 100 μL of an SM2 buffer solution containing P4-EKORhE transducing particles harbouring the multi-lysin cassette was supplemented after 1 hour of bacterial incubation on the A549 cell layer and another where 100 μL of blank SM2 buffer was added instead. The figure reveals that the differences in bacterial count after 1 hour of bacterial growth, followed by 2 hours post-treatment with P4-EKORhE, indicate the system’s ability to deliver the multi-lysin cassette in a complex environment. **Figure 7D** shows a remarkable difference in average bacterial counts between samples treated with P4-EKORhE particles in SM2 buffer (target samples) and those treated with SM2 buffer only (negative control). However, the significance analysis for a sample size (n) of n = 6 yields only p = 0.31, suggesting the need for further optimisation or larger sample sizes to substantiate the observed differences. The images in **Figure 7E** further illustrate this remarkable difference in averages, and additional images used for bacteria/cell nuclei counts are available in **Supplementary Figure S13** for target samples and **Supplementary Figure S14** for negative controls.

In the context of evaluating the multi-lysin cassette as a gene-delivered antimicrobial, these results demonstrate the potential of P4-EKORhE as a high-yield transducing particle bioproduction system capable of delivering a genetically encoded antimicrobial (the multi-lysin cassette) in a human cell culture infection model, thereby corroborating the results obtained from pure bacterial cultures.

## 3 Discussion

### 3.1 Effectiveness of the multi-lysin cassette

In regard to the effectiveness of the multi-lysin cassette, **Figure 4** suggests that the activation of lysis leads to an antimicrobial effect that is shown over the period of 5 hours, which suggests this device as a powerful alternative to natural phages because of the observed decrease in bacterial cell optical densities.

The results demonstrated in **Figure 4** indicate that the *λ*-lysis cassette and MS2 gpL have moderate but detectable activities even before induction, likely due to the accumulation of gene products (LysS holin/antiholin, Rz and Rz1 spanins, and the LysR endolysin) over time. Both MS2 gpL and PhiX174 gpE appear only in aTc-induced samples, consistent with the absence of cryptic promoters or SD sequences upstream of their respective coding sequences (**Supplementary Figure S8**). The constitutive expression of LysS and LysR proteins, as well as Rz, can be explained by the presence of a cryptic promoter upstream of the potential SD-like sequence “GGAAGGAG” at the −3 position of PhiX174 gpE, or from upstream cryptic promoters in the sequences *lysS* and *Rz* (found exclusively in non-induced samples) (**Supplementary Section SS8**). **Figure 4B** confirms cryptic promoters and SD sequences, evidencing their presence when the cosmid is not activated with aTc. However, due to the minimal contribution of these cryptic elements (especially for PhiX174 gpE), they do not impede the system’s use for transducing particle production, as shown in **Figure 5C**. In contrast, MS2 gpL and PhiX174 gpE are only present in aTc-induced samples, supporting their reliance on induction.

The hypothesised mechanism for the synergistic lysis dynamics of the multi-lysin cassette is as follows (refer to **Supplementary Section SS4.5** for a comprehensive discussion): 1) *λ*’s LysS (UniProt Consortium, 2024d) accumulates in the cytoplasm and, upon reaching critical concentration, forms pores in the cytoplasmic side of the bacterial membrane (White et al., 2010). 2) This pore formation facilitates the intrusion of *λ* LysR endolysin (White et al., 2011; UniProt Consortium, 2024c), along with single-gene lysins MS2’s gpL (UniProt Consortium, 2024b) and PhiX174’s gpE (UniProt Consortium, 2024a), which disrupt the membrane (Bernhardt et al., 2002; Orta et al., 2023). 3) *λ*’s Rz (UniProt Consortium, 2024e) and Rz1 (UniProt Consortium, 2024f) then deliver the final blow after the membrane is disrupted (Zhang and Young, 1999; Berry et al., 2012). This lysis is observed in non-aTc-induced (non-induced) samples (from cryptic promoter/SD sequences allowing *λ*-lysis cassette expression), suggesting an automatic activation of lysis when the accumulation of gene products occurs. The results confirm that the activation of the multi-lysin cassette with aTc corresponds to the presence of lysins, lysis accessory proteins, and antimicrobial activity as shown in **Figure 4**.

Regarding the effectiveness of using lysins, this work demonstrates that the multi-lysin cassette—encoding a combination of lysins and lysis accessory proteins (such as holins and spanins)—constructs a genetically encoded antimicrobial with synergistic effects by activating multiple lysis mechanisms. The results obtained suggest that the incorporation of additional kill switches, such as nuclease elements (e.g. CRISPR-Cas9) (Singh et al., 2017) or alternative growth inhibitors that target divergent bacterial survival pathways, could further enhance the cassette’s potency.

### 3.2 High-yield bioproduction with P4-EKORhE

The assessment of the capacity of P4-EKORhE for the production of transducing particles, shown in **Figure 5D**, depicts the dynamics of P4-EKORhE carrying the multi-lysin cassette. The data show that this production system represses phage production when rhamnose is absent (while maintaining 50 μg/mL kanamycin in the culture) and activates fully when 0.2% (w/v) rhamnose is added. This work highlights the characteristic feature of P4-EKORhE for controllable propagation (**Figures 5**, **6**, **8**), which is a milestone for bioproduction because 1) it enables the delay in the activation of phage bioproduction during the exponential phase to maximise bacterial cell densities and thus phage yields and 2) it forcefully activates phage bioproduction through *ϵ* inducible heterologous expression. As expected, the P4-EKORhE propagates only in Δcos:TriR-P2-c5545 Z1 cells since no *ϵ* gene activation was observed in DH5*α* Z1 cells upon rhamnose treatment. Instead, P4-EKORhE remains repressed in the absence of rhamnose and becomes fully inducible when 0.2% (w/v) rhamnose is added. Moreover, a notable drop in OD_600_ was observed upon the addition of aTc to the production culture due to the controlled activation of the multi-lysin cassette. **Figure 5D** demonstrates that although repression of P4-EKORhE is not absolute, it sustains a stable culture suitable for high-yield bioproduction. This stability allows for the maintenance of P4-EKORhE, alongside other cosmids, within a production strain, such as Δcos:TriR-P2-c5545 Z1, using a transformation protocol and to store the strain in glycerol stocks at −80°C for later expansion. The frozen strain can then be used as a master seed for future culture expansion and further induced with 0.2% (w/v) rhamnose to produce a new batch of P4-EKORhE transducing particles. In this way, the repressible capability of P4-EKORhE allows for an easier and more reproducible production of P4-based transducing particles. Conclusively, the bioengineered P4-EKORhE, a minimised, controllable, and conditionally propagating phage that employs temperate P2 phages with *knocked-out* cos sites, produces virus-free P4-like transducing particles at remarkably high yields (ranging from 10^6^ to 10^10^ pfu/mL) while eliminating infectious wild-type P2 or P4 phages. The *ϵ* gene was used as a transactivator to exploit natural P4 phage proteins that activate temperate P2 phages, thereby enabling the reproducible and controllable production of transducing particles. The further downstream processing optimisations (using nutrient-rich TB and an optimised hybrid fed-batch process) achieve yields greater than 10^12^ pfu/mL, demonstrating that the P4-EKORhE system provides an optimal procedure for the high-yield production of transducing particles.

### 3.3 Transduction of the multi-lysin cassette with P4-EKORhE

**Figures 7A–C** confirm the initial characterisation of the multi-lysin cassette in **Figure 4**. Notably, increasing the MOI from 10 to 100 for natural phages did not produce an appreciably different result, even though an MOI of 10 produces transient growth at approximately 1 hour post-infection, while P4EKORhE delays growth until after 2.5 hours, as reflected in the relative area under the curve (rAuC). Moreover, at an MOI of 100, the antimicrobial activity in negative control strains of *E. coli* Marionette, where the tetR repressor remains active in the absence of aTc, suggests an additional mechanism involved in the lysis that goes beyond the expression of lysins.

To facilitate discussion, **Figure 7C** uses the rAuC for the growth curves in **Figures 7A, B** to compare antimicrobial effectiveness among samples and MOIs for both P4-EKORhE and T4/K1F-GFP phages. These results show that infecting *E. coli* BW25114 with T4 produces a response similar to that observed with controllable P4-EKORhE. Of note, K1F phages specialise in infecting encapsulated strains of *E. coli* (Møller-Olsen et al., 2018), so their effectiveness against normal strains such as BW25113 is expected to be compromised, as shown by the figure and corresponding rAuC values.

Regarding the effect of MOI on the bacteriostatic activity of P4-EKORhE encoding the multi-lysin cassette, **Figure 7A** and literature (Abedon, 1994) indicate that at high MOIs (100), “lysis-from-without” may contribute to the observed results, although the multi-lysin cassette plays the primary role at lower MOIs (10). This effect becomes apparent in the negative control results at MOIs of 100 without aTc. **Figure 7C** shows that the differences in rAuC values for MOIs of 10 versus MOIs of 100 with P4-EKORhE do not scale equally between BW25113 and the Z1 controls, suggesting that “lysis-from-without” does not fully account for the differences. However, the non-induced Z1 strains display a notable increase in antimicrobial effectiveness only at an MOI of 100, which may result from “lysis-from-without” if the tetR repressor remains fully functional in the absence of aTc. An alternative, although less likely, would be the effect of tetR repressor depletion. At high MOIs (e.g. 100), a single bacterial cell may be transduced multiple times, exhausting its tetR repressors and leading to leaky gene expression of the multi-lysin cassette. This phenomenon could contribute to the bacteriostatic effect observed at high MOI but is not apparent at lower MOIs of 10 (compare the rAuC and the bacteriostatic times for the “Z1 + aTc” versus the “Z1 non-aTc”, when using the P4-EKORhE, in the rAuC plot in **Figure 7C**).

Whether high MOIs cause effects via “lysis-from-without” or by tetR repressor depletion remains speculative. Further experiments, for example, using P4-EKORhE carrying blank genetic sequences, could clarify this contribution. Based on these data, it is possible to conclude that the substantial differences between aTc-induced and non-induced Z1 cells cannot be solely attributed to the multi-lysin cassette, as antimicrobial effectiveness (albeit milder) at low MOIs (10) is still observed, which is less pronounced than in the “Z1 Marionette + aTc” positive control.

If “lysis-from-without” occurred at lower MOIs, the 2.5-hour lysis delay in the negative control (**Figure 7A**) would not be observed. In both replicas (**Figure 7A** and **Supplementary Figure S2**) for non-activated Z1 cells infected with P4-EKORhE, a mild antimicrobial activity at an MOI of 100 (indicated by a bump in growth at 2.5 hours starting at time = 0), which suggests that the effect in the negative controls at high MOIs likely results from “lysis-from-without” or tetR repressor depletion, was observed.

These data underscore the potential of P4-EKORhE particles as an antimicrobial alternative to natural phages. **Figures 7B, C** show that BW25113 and Z1 Marionette, when infected with T4 phage, fail to grow during the 5-hour measurement period, similar to transduction with P4-EKORhE. These results support the feasibility of using transducing particles as an alternative to natural phages, with the advantage of full control over lysis mechanisms and without relying on viral replication in target bacteria.

The transduction of the multi-lysin cassette via P4-EKORhE produces an antimicrobial effect that is not compromised but rather enhanced compared to the results obtained in transformed bacterial cell cultures. The results obtained from pure bacterial cultures closely resemble those found with natural phages such as T4, highlighting the clinical potential of both P4-EKORhE and the multi-lysin cassette. Moreover, these data indicate that antibiotic marker selection (used to maintain the transduced cosmids within bacterial hosts) does not impair performance at MOIs of 10 or 100, suggesting that antibiotic co-delivery is unnecessary for achieving similar or superior bacteriostatic effects compared to systems where kanamycin (50 μg/mL) is added before aTc activation. An improved antimicrobial effect can be attributed to multiple transducing particles that likely transduce cosmids multiple times during the initial hours of infection. Additionally, at high MOIs (100), the phenomenon of “lysis-from-without” may enhance the effect, as indicated by comparisons between positive and negative control outcomes with P4-EKORhE. The use of P4-EKORhE harbouring the multi-lysin cassette in A549 cell cultures contaminated with *E. coli* shows (despite a p-value of 0.31) a notable reduction in average bacterial counts that correlates with results from pure bacterial cell cultures. The findings invite further experiments at higher MOIs, with additional replicas and testing on different cell lines, where a clearer difference between treated and untreated samples may be observed, especially considering that P4-EKORhE does not propagate in target host bacteria.

Ultimately, these data demonstrate that encoding the multi-lysin cassette in a cosmid packaged within a P4-EKORhE system produces a non-replicative, yet conditionally propagable, finely tunable antimicrobial agent as effective as natural phages. Therefore, this work provides a comprehensive proof-of-concept gene therapeutic where a phage-based transducing particle with a genetically encoded antimicrobial offers an alternative to natural phages using P4-EKORhE as gene delivery vehicle to produce high yields of transducing particles for delivering the multi-lysin cassette into target bacteria—both in pure bacterial cultures and in co-cultured using A549 human epithelial cell cultures infected with *E. coli*. This approach opens the door to novel and effective antimicrobial therapeutics targeting a range of pathogenic bacterial strains.

## 4 Materials and methods

### 4.1 General materials

All chemicals were purchased from Sigma-Aldrich (now Merck, Kenilworth, NJ, USA) or Thermo Fisher Scientific (Waltham, MA, USA) unless stated otherwise. Primers and gene fragments were commercially synthesised by Integrated DNA Technologies (IDT; Coralville, IA, USA) or GENEWIZ (South Plainfield, NJ, USA). The stock and working concentrations of antibiotics used in this work are stated in **Supplementary Table S1**. All strains used throughout this work are stated in **Supplementary Table S3**. For the construction of genetic devices (**Supplementary Section SS4** and **SS8**), Q5 High-Fidelity Polymerase, Phusion High-Fidelity DNA Polymerase, and NEBuilder HiFi DNA Assembly Master Mix were purchased from New England Biolabs (NEB; Ipswich, MA, USA) and used according to protocol. All enzymes were stored at −20°C and used with the buffers provided according to the manufacturer’s instructions.

### 4.2 Mass spectrometry and proteomics

In order to ascertain that bacterial cells are eliminated upon the activation of the multi-lysin cassette when using aTc, it is useful to identify these lysins on via Western blotting only when the circuits are activated in order to relate the correspondence between gene expression and a decrease in the optical cell densities, as shown in **Figure 4**. However, because the encoded lysins included in the multi-lysin cassette are many (i.e. up to six different coding sequences, **Supplementary Figure S12**), when constructing the system, it was unfeasible to include *Histidine-tags*, which would otherwise facilitate its purification and identification because having repetitive DNA sequences within the cassette would generate genetic stability problems in the cosmids encoding repetitive sequences, which would lead to the impossibility of gene synthesis using a commercial supplier. Therefore, in order to demonstrate the presence of the lysins, upon activation with aTc, and to relate its expression to its antimicrobial effect, an sodium dodecyl sulfate polyacrylamide gel electrophoresis (SDS-PAGE) gel for expression verification (Protocol SS6.12), and later extraction for mass spectrometry (MS) analysis, was employed after the protein extraction of experimental samples (Protocol SS6.11). However, because bacterial cells would be disrupted upon the activation of the lysin cassette, an SDS–PAGE gel did not yield bands sharp enough to be distinguished by raw inspection (i.e. to distinguish them among the remaining proteins in the lysate). Instead, a lower fraction band of the SDS–PAGE gel was used for protein extraction and MS, confirming the presence of the lysins and lysis accessory proteins showcased in this work (**Figure 4**). To prepare samples for MS (**Supplementary Section SS3**), induced and non-induced protein lysates from a culture of Marionette Z1 bacterial cells containing a P4-EKORhE cosmid, i.e. in **Figure 5**, which harbours the aTc-inducible multi-lysin cassette showcased in this work, was processed and purified using a standard protein extraction protocol (see Protocol SS6.11). Two types of lysates were produced, one coming from the pellet, or insoluble phase (i.e. as lysins are expected to act within cell walls and membranes), and another that was considered a “raw lysate”, i.e. containing both soluble and insoluble phases of the lysate. Four different samples of pure bacterial cell cultures (*E. coli* Z1 cells) transformed with the multi-lysin cassette cosmid were grown in a 1-mL Eppendorf tube at 37°C under shaking conditions until reaching the exponential growth phase (approximately 0.4–0.5 OD_600_). Subsequently, two of the samples were aTc-induced with 200 nM (Zhang and Poh, 2018) of anhydrous tetracycline and grown for a further 2 hours to allow for the lysis of the bacterial cell culture. The bacterial cell cultures were treated with Lysis buffer (50 mM Tris-HCl, 150 mM NaCl, 1% Triton X-100, and 5 mM EDTA) and sonicated to make sure both induced and non-induced samples were treated equally and also so that non-induced samples were indeed lysed (see Protocol SS6.11 for more details).

### 4.3 Preparation of optical density measurements

In order to ascertain the antimicrobial effectiveness of the P4-EKORhE transducing particles encoding the multi-lysin cassette (**Figures 7A, B**), replicas of 96-well plates were utilised. For each well on a 96-well plate (Cellstar Multiwell Plates, Sigma-Aldrich), 100 μL of bacterial culture of *E. coli* at an OD_600_ = 0.1 was dissolved in doubly concentrated LB media and half diluted with an additional 100 μL of either an SM2 buffer solution supplemented with P4-EKORhE multi-lysin transducing particles or T4 or T7 phages (at MOIs of 10 or 100) or with an SM2 buffer only solution for blanks (i.e. for MOI = 0). The plates were measured using a plate reader to measure optical densities at approximately 600 nm to determine bacterial cell densities over time. When the bacterial cells being tested were the Z1 cells, and there was the intention to activate the multi-lysin cassette upon infection with P4-EKORhE, anhydrous tetracycline was added at a concentration of 200 nM (Zhang and Poh, 2018). For the transformed multi-lysin cassette experiments (**Figures 4**, **5D**), the same methodology was employed, but without doubling LB medium concentration and without adding any SM2 buffer solutions, kanamycin (50 μg/mL) was used instead to recover the cells after cosmid transformation and selection before activation. Before activation, cosmid-harbouring cells were resuspended in LB media, the multi-lysin cassette was activated using anhydrous tetracycline (aTc, 200 nM) (Zhang and Poh, 2018), and l-rhamnose was added at a concentration of 0.2% (w/v) (Wegerer et al., 2008).

### 4.4 Relative area under the curve as antimicrobial effectiveness

For a more accurate comparison between the antimicrobial effect of the P4-EKORhE transducing particles harbouring the multi-lysin cassette with the antimicrobial effect of natural phages T4 and K1F-GFP, the rAuC values from the OD_600_ plots over the time span of 0 to 5 hours were extracted for all plots. The rAuC was plotted using the area under the curve (AuC) obtained using the scripted def AuC(x, y): function shown below of the non-induced or non-transduced (i.e. MOI = 0) sample and using it to subtract the mean values of the induced samples and the standard deviation normalised (by dividing its values) from the blank experiments with non-transduced or non-infected bacterial cells. It is understood that rAuC(MOI = 0) = 0 ± 1, i.e. for MOI = 0, the rAuC = 0 with an absolute standard deviation of 1. Therefore, MOIs of zero were not included in the rAuC plots, as these are a relative measure. For the integration of each of the curves from the corresponding plots used to obtain the values of AuC, which can later be used to compute the rAuC as aforementioned, the trapezoid rule was used to interpolate the area under the curve using the equation encoded in the following script:

0  def AuC(x,y):

1     sum=0

2       for i in range (1, len(x))

3         h=x[i]-x[i-1]

4       sum+=h*(y[i−1]+y[i])/2

5       return sum

where x is the time and y is the point value of OD_600_ over time, which form the basis of the total sum of the area under the curve against the x-axis from x = 0 (i.e. time = 0) until the final value of x.

### 4.5 Preparation of animal tissue cultures for transduction

In regard to the mammalian cell culture for the transduction of co-cultured target bacteria, cell lawns of A549 cells (A549—ATCC CCL-185) were cultivated. The A549 cells were cultured using a base medium F12/K (Gibco, Grand Island, NY, USA/Invitrogen, Carlsbad, CA, USA) with 10% foetal bovine serum (FBS) added to the base medium with a standard amount of penicillin/streptomycin (P/S), incubated at 37°C with 5% CO_2_. The A549 cells were seeded in T25 flasks until they were transferred to 6-well plates (Cellstar Multiwell Plates, Sigma-Aldrich). When the growing A549 cells were transferred to the 6-well plates, using 2 mL of media per well, these were grown on top of microscopy coverslip slides that have been deposited on each of the 6-well plates so as to be able to remove the coverslip slides for later preparation, following an immunohistochemistry protocol, for fluorescence imaging using DAPI, phalloidin, and a green fluorescent protein (GFP) enhancer to count bacterial cells (i.e. transformed with a GFP plasmid) alongside cell nuclei (stained with DAPI) surrounded by the cell cytoskeleton (stained with phalloidin). Once the 6-well plate with the lawn of A549 cells was grown to approximately 90% confluency, the media were changed with 2 mL Leibovitz CO_2_-supplemented media (L-15 Medium, Sigma-Aldrich) with 10% FBS only, without antibiotics, before adding bacteria to the media. Following it, 100 μL of bacteria at an OD_600_ of 0.1 was added to the media, grown, and incubated at 37°C for 1 hour. After that, either 100 μL of a particular SM2 buffer solution supplemented with P4-EKORhE transducing particles harbouring the *multi-*lysin cassette at an MOI of 10 or 100 μL of a blank solution of SM2 buffer (i.e. for MOI = 0) was added to each well. Further incubation for 1 hour was applied before removing the media and starting the following immunohistochemistry protocol. For the immunohistochemistry protocol, which was adapted from the literature (Møller-Olsen et al., 2018), the liquid phase of the cell cultures grown in the 6-well plate was removed by aspiration. The 6-well plate was placed under the fume hood, and cells were fixed by adding 500 μL of a solution of 4% paraformaldehyde (PFA) before placing the plate on ice for 15 minutes. The liquid was again removed from the plate, and its wells were washed three times with phosphate-buffered saline (PBS) for 5 minutes each time. The cells were then permeabilised with 500 μL of ice-cold PEM/0.05% saponin [PEM Buffer: 0.1 M PIPES/1,4-piperazinediethanesulfonic acid, piperazine-1,4-bis(2-ethanesulfonic acid), piperazine-*N*,*N*′-bis(2-ethanesulfonic acid), 5 mM EGTA, and 2 mM magnesium chloride], brought to pH 6.8 using a NaOH solution, and later aspirated and washed three times with PBS for 5 minutes. The remaining cells on each well were aspirated before being incubated for 15 minutes, at room temperature, with 500 μL of a 50-mM solution of ammonium chloride. The wells containing the coverslip slides with the cells were then aspirated and washed once for 5 minutes with PBS/0.05% saponin. Subsequently, 40 μL of a mixture that contains 300 μL of 0.05% saponin, 7.5 μL of phalloidin, and 1.5 μL of a GFP enhancer was added to each of the six wells of the plate and incubated for 45 minutes in the dark. The wells were then washed twice for 5 minutes with PBS/0.05% saponin and once with PBS. After aspiration, a drip of Fluoroshield (Fluoroshield™ with DAPI, Sigma-Aldrich) was added to each coverslip glass slide in each well of the 6-well plate. The coverslips were then delicately rinsed with water before being put on microscope slides. The microscope slides with the coverslips were then allowed to dry in the dark, and after sealing with CoverGrip (CoverGrip™, Biotium, Fremont, CA, USA) sealant, they were finally stored at 4°C until microscopy imaging. For the microscopy (ZEISS Celldiscoverer 7—Automated Live Cell Imaging, Oberkochen, Germany), images were taken at random, and images where more than 60 nuclei per image could be counted were taken to ensure the homogeneity of the samples from which imaging was performed. After obtaining the images, bacteria were counted in each image with respect to the number of nuclei (blue nuclei stained with DAPI), together with bacteria expressing GFP (green dots stained with GFP enhancer).

### 4.6 Upstream optimisation and downstream processing with P4-EKORhE

For the production of P4-EKORhE-based transducing particles harbouring the multi-lysin cassette, several protocols were tested, which have led to different results. The outputs of such protocols are shown in **Supplementary Figure S3**.

The proposed protocol was established for producing transducing particles by growing an overnight (O/N) culture of *E. coli* Δcos:TriR-P2-c5545, which was pre-transformed with a P4-EKORhE multi-lysin cosmid in culture media of LB with 50 μg/mL kanamycin (to select for the selection marker in the P4-EKORhE) and 10 μg/mL trimethoprim (to select for the Δcos:TriR cassette in the c5545 strain harbouring the P2 prophage) at 37°C. Then, 10% (v/v) of the O/N culture was combined with LB media using 50 μg/mL kanamycin and 10 μg/mL trimethoprim. The culture was shaken for 3–4 hours at 150 rpm and 37°C and grown until the exponential phase (OD_600_ to 0.2–0.5). During the exponential phase, the epsilon (*ϵ*) gene encoded within the P4-EKORhE cosmid was activated by adding 0.2% (w/v) rhamnose to the media and further incubated at 150 rpm and 37°C for 3 hours until lysis was observed. The debris from the lysate was pelleted at 4,000 *g* for 30 minutes. The lysate was then filter-sterilised, with the option to store the filter-sterilised lysates at 4°C if desired.

After the lysate was obtained, a PEG8000 concentration protocol (Protocol SS7.2) or an ultrafiltration protocol (Bonilla et al., 2016) was applied in order to purify and concentrate the lysate samples with the conditionally propagating transducing particles. When using the PEG8000 protocol, the yields would not improve over 10^6^ transducing particles per millilitre (**Supplementary Figure S3**, measured as pfu/mL). An alternative strategy using ultrafiltration is shown to improve the yields to up to 10^10^ particles per millilitre (as can be seen in the replica “P4-EKORhE, ×1 LB broth + ultrafiltration” shown in **Supplementary Figure S3**).

Fortunately, even higher yields of P4-EKORhE transducing particles were obtained when using TB as culturing media and using a four-step enrichment technique, followed by ultrafiltration (as depicted in **Figure 8**), with observed yields of up to 10^12^ particles per millilitre, as it is seen in the three replicas on the top row of the spot assay shown in **Figure 6**.

The protocol, using the showcased enrichment technique, is characteristic of P4-EKORhE because of its capacity to be repressed and activated (i.e. a controllable P4-EKORhE system). This characteristic allows for the multiplication of the Δcos:TriR-P2-c5545 Marionette Z1 *E. coli* lysogenic bacteria in a *fed-batch* fashion while maintaining a “dormant cosmid” before its activation with 0.2% (w/v) rhamnose. A high concentration of producing bacteria harbouring the P4-EKORhE is thus possible, and the later activation of the P4-EKORhE renders the highest yields obtained throughout this work. In this procedure, an O/N culture of Δcos:TriR-P2-c5545 *E. coli* lysogens, previously transformed with the P4-EKORhE cosmid, was grown in LB medium using 50 μg/mL kanamycin (to select for the selection marker in the P4-EKORhE) and 10 μg/mL trimethoprim (to select for the Δcos:TriR cassette in the c5545 strain harbouring the P2 prophage). Subsequently, 10% (v/v) of the aforementioned overnight culture was combined with fresh LB media (with kanamycin and trimethoprim) and shaken for 2 hours at 150 rpm and 37°C until the culture was recalled at the exponential growth phase (OD_600_ = 0.5). Concurrently, another 4% (v/v) portion of the overnight culture was pre-mixed with the necessary amount of CaCl_2_ to make a final solution of 0.1 M in a flask containing a volume of TB media, including kanamycin and trimethoprim at the aforementioned concentrations, and shaken for 3–4 hours at 150 rpm and 37°C, reaching the exponential growth phase. Pre-mixture of cells with CaCl_2_ is essential to avoid the precipitation of phosphate salts in the TB broth used for the culture. After reaching the exponential growth phase, 0.2% (w/v) rhamnose was added to the TB culture to induce the production of the *ϵ* gene to activate the Δcos:TriR-P2 prophage. After 2 hours of lysis in the TB culture, a 4% (v/v) sample of bacteria from the LB culture was introduced into the lysing TB-grown culture, again pre-mixing it with the necessary amount of CaCl_2_ to maintain a final solution of 0.1 M in the growing TB culture and compensating the rhamnose to maintain the 0.2% (w/v) proportion. Nevertheless, concurrently, a new exponentially growing LB culture was prepared by adding 10% (v/v) of the overnight culture, which was shaken in parallel for a further 2 hours at 150 rpm and 37°C until it reached the “recall stage” at the exponential growth phase. This growing LB culture was also introduced into the ongoing lysis process of the TB culture, after 2 hours, and pre-mixed with the necessary amount of CaCl_2_ to make a final solution of 0.1 M while maintaining the proportion to a final 0.2% (w/v) rhamnose. This re-lysing procedure can be repeated as many times as necessary (e.g. 0 to 3 times), ideally up to three additional times, to maximise the yield of transducing particles obtained. The lysis procedure was finished by adding 0.1 M MgCl_2_, and the culture was shaken at 37°C for a further 1.5 hours until visible clumps were observed. Afterwards, the debris from the lysate was pelleted at 4,000 *g* for 30 minutes, followed by filter sterilisation of the lysate using 0.22-μm sterile syringe filters. After filtration, the lysate can be paused and stored at 4°C if desired until further use. The lysate can then be ultrafiltrated using the Amicon 100-kDa cut-off columns by centrifuging at 3,000 *g*. The recovered, non-eluted residue contains the highly concentrated P4-EKORhE-based transducing particles. Before titration, the lysate was pre-treated with DNAseI from NEB using 10 μL lysate + 10 μL DNAseI buffer + 10 μL DNAseI + 79 μL of water and incubated for 1 hour. Because P4 phages were conditionally propagated, lysates were titrated using a spot assay (**Supplementary Section SS7.4.1**) on lawned plates of Δcos:TriR-P2-c5545 Marionette Z1 *E. coli* lysogenic bacteria with 5 mM Ca^++^.

## Data Availability

The raw data supporting the conclusions of this article will be made available by the authors upon reasonable request.
